# Hearing assistance technologies and social health among individuals with care and support needs: a scoping review

**DOI:** 10.1186/s12877-026-08681-3

**Published:** 2026-11-10

**Authors:** Bugse Yüceer, Charlotte Janning, Seda Güney, Karin Wolf-Ostermann

**Affiliations:** 1 Institute of Public Health and Nursing Research (IPP), University of Bremen, Bremen, Germany; 2 Carl von Ossietzky Universität Oldenburg, Oldenburg, Germany; 3 School of Nursing, Koç University, Istanbul, Türkiye

**Keywords:** Hearing assistance technologies, Hearing aids, Hearing loss, Social health, Social participation, Care and support needs, Older adults

## Abstract

**Background:**

Hearing loss can affect communication, social interaction, participation, and the maintenance of social relationships, particularly among individuals who require care or support in everyday life. Hearing assistance technologies (HATs) may facilitate communication, but their relationship with the multiple dimensions of social health remains insufficiently understood. A multidimensional social-health framework encompassing Capacity, Independence, Social Participation, Structure, Function, and Appraisal provides a basis for examining these relationships beyond individual hearing outcomes. This scoping review aimed to map the existing evidence on HATs in relation to social health among individuals with care and support needs.

**Methods:**

This scoping review followed the PRISMA-ScR guidelines and the methodological framework proposed by Arksey and O’Malley and further refined by Levac. PubMed, CINAHL, Scopus, the Cochrane Library, Ovid MEDLINE, Web of Science, and IEEE Xplore were searched. Studies were eligible if they included individuals aged ≥ 18 years with care and support needs and examined HATs in relation to findings relevant to social health. Data were charted and synthesised descriptively, and findings were organised according to the six domains of the multidimensional social-health framework, comprising Capacity, Independence, Social Participation, Structure, Function, and Appraisal.

**Results:**

The searches identified 1,145 records, of which 21 studies met the inclusion criteria. The included samples were predominantly older. Findings were most frequently mapped to the Appraisal and Social Participation domains, whereas Structure and Function were comparatively underrepresented. Intervention findings included improvements in communication-related outcomes and reductions in hearing-related participation restrictions, whereas findings for loneliness, relationship satisfaction, social-network characteristics, and broader social engagement were less consistent. Qualitative findings highlighted stigma and perceived social judgement, with the social acceptability of hearing technologies also described in relation to everyday interaction and technology use.

**Conclusions:**

Evidence on HATs in relation to social health among individuals with care and support needs remains heterogeneous and uneven across social-health domains. Limited attention to the structural and functional dimensions of social relationships highlights the need for more comprehensive assessment of social health in hearing-care research. Future studies should adopt longitudinal, network-sensitive, and culturally responsive approaches and consider the interpersonal, environmental, and care contexts in which HATs are used.

**Supplementary Information:**

The online version contains supplementary material available at https://doi.org/10.1186/s12877-026-08681-3.

## Background

Hearing loss is a highly prevalent health condition worldwide and represents a major public health concern [1]. More than 1.5 billion people are estimated to live with some degree of hearing loss, and this number is expected to increase substantially over the coming decades [2, 3]. Its implications extend beyond auditory perception and become particularly relevant when hearing difficulties intersect with everyday care and support [4]. Care and support needs may arise across multiple areas of everyday life, including daily activities, mobility and self-care, communication, decision-making, social relationships and participation, and engagement with health and care services [4, 5]. Their nature and extent vary according to the interplay between individual capacities, everyday demands, and the social and environmental resources available to the person. Such needs should not be inferred from age, disability, chronic illness, or hearing loss alone, but from the support required within a person’s particular functional, relational, and environmental circumstances. Formal and informal support can involve assistance with daily activities, communication, or decision-making and can be provided through family and social networks, health and social-care professionals, community services, or residential care arrangements [5]. Support is embedded within social relationships and can enable individuals to remain involved in decisions, everyday activities, and valued social roles while maintaining reciprocal connections with others [6, 7]. This perspective recognises care and support as relational processes that may involve interdependence and reciprocity rather than dependency alone [7]. Hearing-related communication is therefore closely connected to how care, support, participation, and relationships are negotiated in everyday life [8].

Because these processes depend substantially on interaction, hearing-related communication can be understood as relational, shaped by the interplay between auditory capacity, communication partners, and the conditions in which interaction takes place. Background noise, multiple speakers, limited visual cues, reverberation, or rapidly changing conversations may increase the demands of listening and require repetition, clarification, greater listening effort, or conversational repair [1, 8, 9]. Communication partners may respond by modifying speech, rephrasing information, adjusting their position, or altering features of the environment, thereby contributing to how communication is negotiated and sustained [9, 10]. Evidence from everyday listening and communication-partner research indicates that hearing-related difficulties may extend to shared activities, patterns of interaction, and opportunities for social engagement rather than being experienced solely at the level of auditory perception [9, 10]. These processes are especially pertinent in care and support contexts, where communication is integral to understanding and conveying information, coordinating assistance, participating in shared decisions, and maintaining relationships with family members, care partners, and professionals [10, 11].

The relational character of hearing-related communication is particularly relevant to understanding its social-health dimensions. Hearing loss has been associated with changes in social participation, social interaction, social connectedness, and experiences of loneliness and social isolation [12–14]. Social health provides a multidimensional perspective on these experiences by considering both the capacities through which individuals participate in social life and the resources available within their immediate social environment [15–17]. This conception is consistent with broader understandings of health as the capacity to adapt and self-manage in response to physical, emotional, and social challenges [18]. Within the framework operationalised and subsequently extended by Vernooij-Dassen and colleagues, individual dimensions of social health include capacity, independence, and social participation, while the social environment is represented through the structure of social networks, the functions provided through relationships, and the appraisal of the quality of relationships and interactions [16, 17, 19]. Social-network characteristics such as network size, composition, and frequency of contact, supportive functions such as emotional or instrumental assistance, and relational appraisal through experiences such as relationship satisfaction and loneliness therefore represent conceptually distinct but complementary components within the multidimensional social-health framework [17, 19]. This structure provides a basis for organising findings across distinct but interrelated dimensions of social health [17].

Although this framework was developed principally in the context of dementia, its underlying structure centres on the interaction between individual capacities and social-environmental resources rather than on features specific to a single diagnostic condition [17]. This person–environment orientation is also relevant to hearing-related social functioning, where communication, independence, and participation are shaped by auditory capacity in conjunction with communication partners, opportunities for interaction, available support, and environmental accessibility [9, 14]. Accordingly, the framework provides a conceptual lens for examining hearing-related social outcomes across individual and social-environmental dimensions, while retaining the original meanings of its domains.

Hearing assistance technologies (HATs) encompass a range of approaches aimed at improving access to auditory information and supporting communication [20–22]. These include conventional hearing aids, assistive listening systems and personal amplification devices, connected hearing technologies, and emerging hearable devices. Improved access to speech and other auditory information may facilitate conversational engagement and participation [23–25]. Whether these changes are reflected in everyday social life, however, depends on more than auditory access alone [11, 26]. Device suitability and usability, individual communication priorities, communication-partner practices, affordability, access to assessment and follow-up services, and the accessibility of physical and social environments may all shape how HATs are incorporated into everyday interactions [8, 11, 14, 23, 24, 26]. HATs can therefore be situated within a broader communicative context in which users, communication partners, technologies, services, and environments jointly contribute to opportunities for communication and participation [14, 26].

Existing evidence syntheses have addressed several components of this relationship. Reviews involving older populations have examined social participation, loneliness, and social isolation in relation to hearing loss and hearing interventions, identifying communication-related difficulties alongside social, behavioural, and environmental factors associated with participation [14, 27, 28]. Broader syntheses across hearing-loss populations have evaluated validated social outcomes associated predominantly with hearing aids and cochlear implants, while evidence from clinical care settings has examined personal amplification technologies specifically in relation to patient–provider communication [11, 29]. Collectively, this literature establishes a growing evidence base concerning the social dimensions of hearing loss and hearing rehabilitation while remaining distributed across particular technologies, population groups, care settings, and selected social outcomes.

However, the extent to which the broader range of HATs, including emerging hearable technologies, has been examined among individuals with care and support needs across the multidimensional domains of social health remains insufficiently characterised. In particular, it remains unclear how evidence concerning capacity, independence, and participation is represented alongside social-network structure and function and the appraisal of social relationships. Given the conceptual and methodological heterogeneity of the literature, a scoping review is particularly appropriate for characterising the extent and nature of the available evidence, examining how social-health domains have been represented, and identifying areas in which evidence remains limited [30, 31]. Accordingly, this scoping review aims to explore the extent and nature of evidence on HATs, including emerging hearable devices, in relation to social health among individuals with care and support needs, interpreted through a multidimensional social-health framework.

## Methods

This scoping review was reported in accordance with the Preferred Reporting Items for Systematic Reviews and Meta-Analyses Extension for Scoping Reviews (PRISMA-ScR) [32]. Its conduct was guided by the framework developed by Arksey and O’Malley [31], further refined by Levac et al. [30], and aligned with updated methodological guidance for scoping reviews provided by the Joanna Briggs Institute [33]. Consistent with this framework, the review followed five interconnected stages: (1) identifying the research question, (2) identifying relevant studies, (3) selecting eligible studies, (4) charting the data, and (5) collating and summarizing the results [30, 31]. To enhance methodological transparency, the review protocol was prospectively registered on the Open Science Framework (OSF) (Registration DOI: https://doi.org/10.17605/OSF.IO/TRK3S). Following protocol registration, the eligibility criteria were further specified to make the exclusion of paediatric populations explicit and to clarify the operational definition of care and support needs used during study selection.

### Study design and research questions

The objective of this scoping review was to examine how HATs, including emerging hearable devices, have been examined in the literature in relation to social health among individuals with care and support needs. The primary research question guiding the review was: *How are HATs described in the literature in relation to social health among individuals with care and support needs?*

To further explore the scope of the evidence, additional questions addressed: (1) the types of HATs studied in this population, (2) the social health outcomes reported in relation to these technologies, and (3) existing gaps in the literature.

### Search Strategy

A comprehensive literature search was conducted across PubMed, CINAHL, Scopus, the Cochrane Library, Ovid MEDLINE, Web of Science, and IEEE Xplore. These databases were selected to ensure coverage across healthcare, social science, and technology-oriented research fields. The search strategy was developed in collaboration with an academic librarian and included a combination of keywords, database-specific controlled vocabulary terms (e.g., MeSH), and Boolean operators related to three core concepts: care and support needs, hearing assistance technologies, and social health. The principal search concepts, structured according to the PCC (Population–Concept–Context) framework, are presented in Table 1.

The electronic searches were completed by 9 April 2025. The complete PubMed search strategy is provided in Additional file 2 to support reproducibility.

Studies published between 1 January 2015 and 31 January 2025 were eligible for inclusion. For articles published online ahead of issue assignment, the first online publication date was used to determine eligibility. Publications in English, German, or Turkish were considered eligible. Following full-text screening, the reference lists of included studies were manually examined to identify additional relevant records through backward citation searching.

**Table 1:** PCC framework and principal search concepts

PCC	Determinant	Search Terms
**P=Population**	Individuals in Need of Care and Support	“need for care” OR “nurs*” OR “care” OR “caring”
**C= Concept**	Hearing Assistance Technologies	Hearing Aids [MeSH] OR “hearing aid*” OR “hearing device*” OR “hearing technolog*” OR “hearing assistive device*” OR “hearing assistive technolog*” OR “assistive listening device*” OR “hearable*” OR “earable*” OR “ear device*” OR “ear sensor*” OR “earbud*” OR “ear-worn*”
	Social Health	“social participation” OR “social health” OR “social isolation” OR “social exclusion” OR “social well-being” OR lonel* OR “community engagement” OR “social engagement” OR “capacity” OR “reciprocity” OR “independence” OR “independent living” OR “autonomy” OR “activities of daily living” OR “decision-making” OR “self-management” OR leisure activit* OR meaningful activit* OR social network* OR “social environment” OR “relationship quality” OR “social relationship” OR community network* OR “social involvement” OR “community involvement” OR “social influence” OR stigma* OR “social interaction*” OR “social control” OR “social support” OR social role* OR social contact* OR “social functioning” OR “interpersonal relations”
**C=Context**	No setting restriction was applied	No context-specific search terms were applied.

Search syntax and controlled vocabulary were adapted to the requirements of each database. The complete PubMed strategy is provided in Additional File 3.

### Eligibility criteria and study selection

Eligibility criteria were developed in accordance with the PCC framework and aligned with the review objective and research questions [33]. Studies were included if they examined individuals with care and support needs, investigated HATs such as hearing aids, hearables, assistive listening or personal amplification devices, or connected hearing technologies, and included outcomes or experiences with identifiable relevance to one or more domains of the multidimensional social-health framework.

In this review, “care and support needs” is used as an umbrella term encompassing needs for care, support, or both. Care and support needs were operationalised in relation to assistance, rehabilitation, adaptation, or compensatory support relevant to everyday communication, functioning, decision-making, or social participation, consistent with broader conceptualisations of care and support as encompassing functional, relational, and environmental dimensions [4, 5]. Evidence of such needs was identified from the primary report through documented communication or functional difficulties or hearing-related handicap; involvement of family members, care partners, or health and social-care professionals; or rehabilitative or compensatory support addressing hearing-related difficulties in everyday life. Chronological age, diagnostic status, disability, chronic illness, or hearing loss in isolation was not considered sufficient to establish care and support needs.

Eligibility was restricted to individuals with care and support needs aged ≥ 18 years. Mixed-age studies were eligible only when data for participants aged ≥ 18 years with care and support needs could be identified separately; studies restricted to paediatric populations and mixed-age studies with inseparable data for eligible participants were excluded. Paediatric hearing care involves developmental and habilitative pathways that differ from the care and support contexts examined in this review [3]. Cognitive impairment and dementia were not exclusionary conditions and were charted as participant characteristics. No restriction was placed on care or living setting. Eligible evidence could arise from private homes and community settings, hearing and other healthcare services, rehabilitation settings, assisted-living or residential environments, and long-term care facilities. Both qualitative and quantitative primary research studies were considered eligible. Peer-reviewed journal articles and full-length peer-reviewed conference proceedings reporting sufficient primary empirical data were eligible. Editorials, opinion papers, protocols, conference abstracts, reviews, commentaries, case reports or case series, theses and dissertations, and non-human studies were excluded. Implantable hearing technologies, including cochlear implants, were outside the predefined HAT scope, as were technologies used exclusively for diagnostic assessment or physiological monitoring. Detailed inclusion and exclusion criteria are provided in Table 2.

**Table 2:** Inclusion and Exclusion Criteria

Inclusion Criteria	Exclusion Criteria
- Studies involving individuals aged ≥ 18 years with care and support needs, evidenced in the primary report by documented communication or functional difficulties or hearing-related handicap; involvement of family members, care partners, or health and social-care professionals; or rehabilitative or compensatory support relevant to everyday communication, functioning, decision-making, or social participation. Mixed-age studies were eligible only when data for participants aged ≥ 18 years meeting these criteria could be identified separately.	- Studies restricted to paediatric populations; mixed-age studies in which data for eligible participants aged ≥ 18 years could not be identified separately; or studies in which care and support needs could not be established from the primary report according to the predefined criteria.
- Studies examining non-implantable HATs, including hearing aids, assistive listening or personal amplification devices, connected hearing technologies, and hearables.	- Studies of cochlear implants or other implantable hearing technologies, or technologies used solely for diagnostic assessment or monitoring.
- Studies reporting quantitative or qualitative findings with identifiable relevance to at least one domain of the multidimensional social-health framework.	- Studies reporting exclusively audiological, cognitive, psychological, physical-health, or technical outcomes without identifiable social-health content.
- Primary quantitative, qualitative, or mixed-methods studies published as peer-reviewed journal articles or full-length peer-reviewed conference proceedings.	- Reviews, editorials, commentaries, opinion papers, protocols, conference abstracts, case reports or case series, theses/dissertations, and non-human studies.
- Studies published between January 2015 and January 2025 in English, German, or Turkish.	- Studies outside the predefined publication period or language criteria.

All identified records were imported into Covidence, where duplicates were removed [34]. Titles and abstracts were screened independently by two researchers (BY, CJ), followed by independent full-text assessment by the same reviewers. Uncertainty regarding eligibility was resolved through discussion against the predefined criteria, with a third reviewer consulted when necessary. Cohen’s kappa was calculated from the reviewers’ independent full-text eligibility decisions before discussion and consensus (κ = 0.83), indicating substantial agreement [35]. Reasons for exclusion at the full-text stage were recorded.

### Data extraction and synthesis of evidence

A standardized data extraction form was developed and piloted on a subset of included studies, consistent with methodological guidance for scoping reviews [36]. Data were independently charted by two reviewers (BY and CJ). Extracted information included publication characteristics, study design, participant characteristics and care and support context, HAT intervention or exposure, measures or data sources, key findings, and findings relevant to social health. Discrepancies in data charting were resolved through discussion and consensus [33, 36].

Findings relevant to social health were mapped deductively onto the multidimensional social-health framework proposed by Vernooij-Dassen and colleagues using the original definitions of its six domains [17]. Capacity refers to the ability to fulfil social potential and obligations, Independence to autonomy in managing everyday life, and Social Participation to involvement in social activities and interactions. Structure concerns the size, composition, and pattern of social ties, Function concerns the support and exchanges provided through relationships, and Appraisal concerns individuals’ perceptions and evaluations of the quality of relationships and interactions [17].

Domain mapping was independently undertaken by three reviewers (BY, CJ, and SG), with disagreements resolved by consensus following discussion. Because some findings corresponded to more than one domain, the same finding could be mapped to multiple domains where conceptually justified. For multidimensional measures, relevant items and subscales were examined where available to clarify the social-health construct represented, particularly where a single instrument encompassed more than one aspect of social functioning [19]. Where only an overall score was reported, interpretation was based on the construct represented by the measure and its conceptual relevance to the original domain definition. Constructs such as communication self-efficacy, hearing-related functioning, device management, stigma, and generic quality of life were interpreted in relation to a social-health domain only when their content and context were consistent with the relevant domain definition; they were not assumed to represent a domain on the basis of the measure or construct label alone. Given the heterogeneity of study designs, populations, technologies, and outcome measures, no quantitative synthesis was undertaken. Cognitive status was retained as a participant characteristic during synthesis because of its potential relevance to communication, HAT management, patterns of technology use, and social-health-related findings [22]. Findings from studies involving cognitive impairment or dementia were interpreted within their specific population and care context. Synthesis also reflected the underlying study design, with observational findings described as associations or observed changes and intervention findings reported according to the comparisons presented in the primary studies.

### Quality appraisal

Consistent with the objectives of a scoping review, formal critical appraisal or risk-of-bias assessment of the included studies was not undertaken, as the purpose of the review was to map the extent, characteristics, and conceptual distribution of the available evidence rather than to estimate comparative effectiveness or establish certainty of intervention effects [33].

**Fig. 1:**
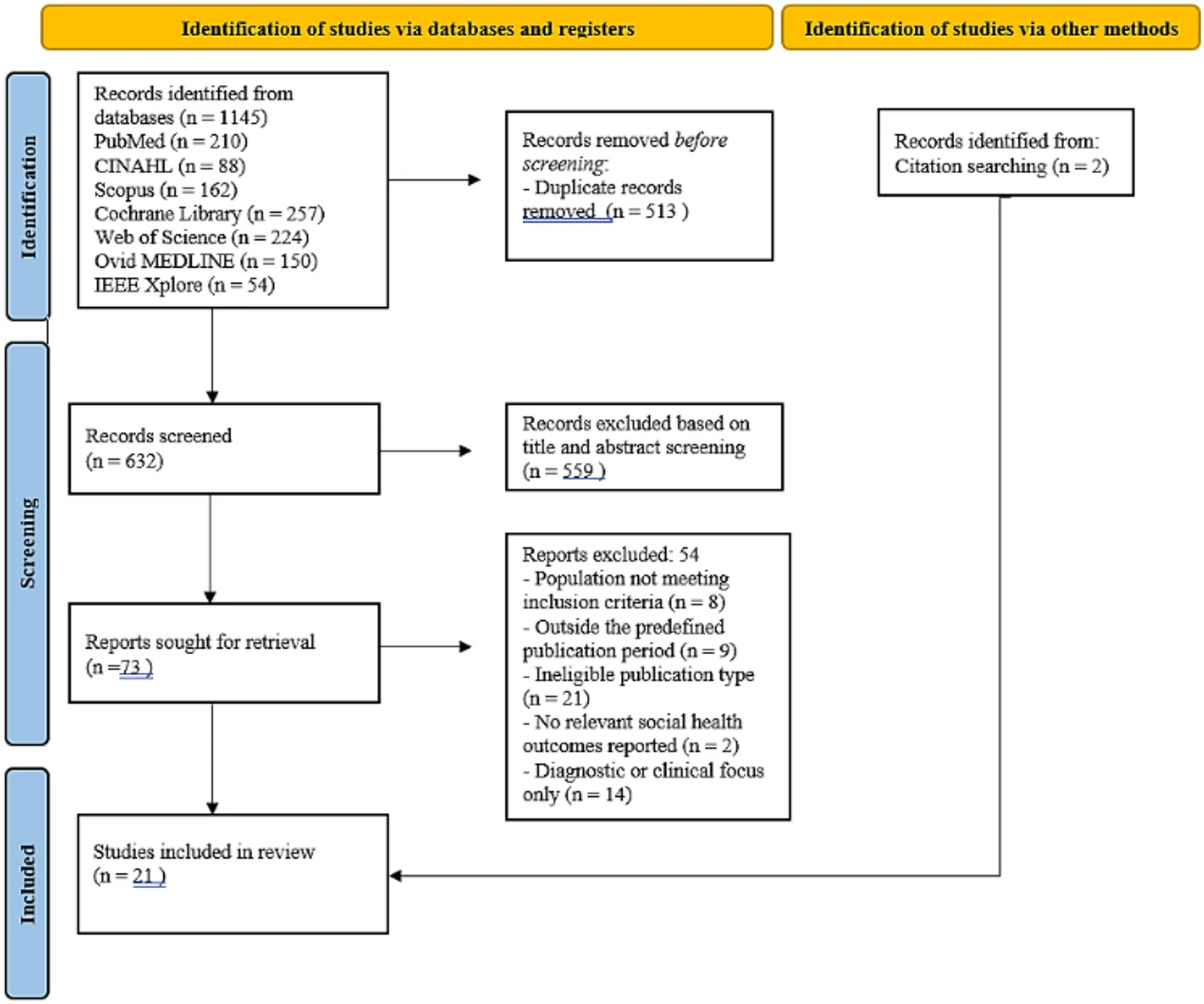
PRISMA flow diagram of study selection

## Results

### Study selection

The database searches identified 1,145 records. After removal of 513 duplicates, 632 titles and abstracts were screened. Seventy-three full-text reports were assessed for eligibility, of which 54 were excluded, leaving 19 studies identified through database searching. Backward citation searching identified two additional eligible studies. In total, 21 studies were included in the review (Fig. 1). Reasons for full-text exclusion included failure to meet the operational population criterion, absence of findings relevant to social health, ineligible publication type or publication period, and exclusive focus on technical or clinical outcomes without social-health relevance. The study selection process is presented in the PRISMA-ScR flow diagram (Fig. 1) [37]. An overview of the characteristics of the included studies is provided in Additional file 1.

### Characteristics of included studies

**Fig. 2:**
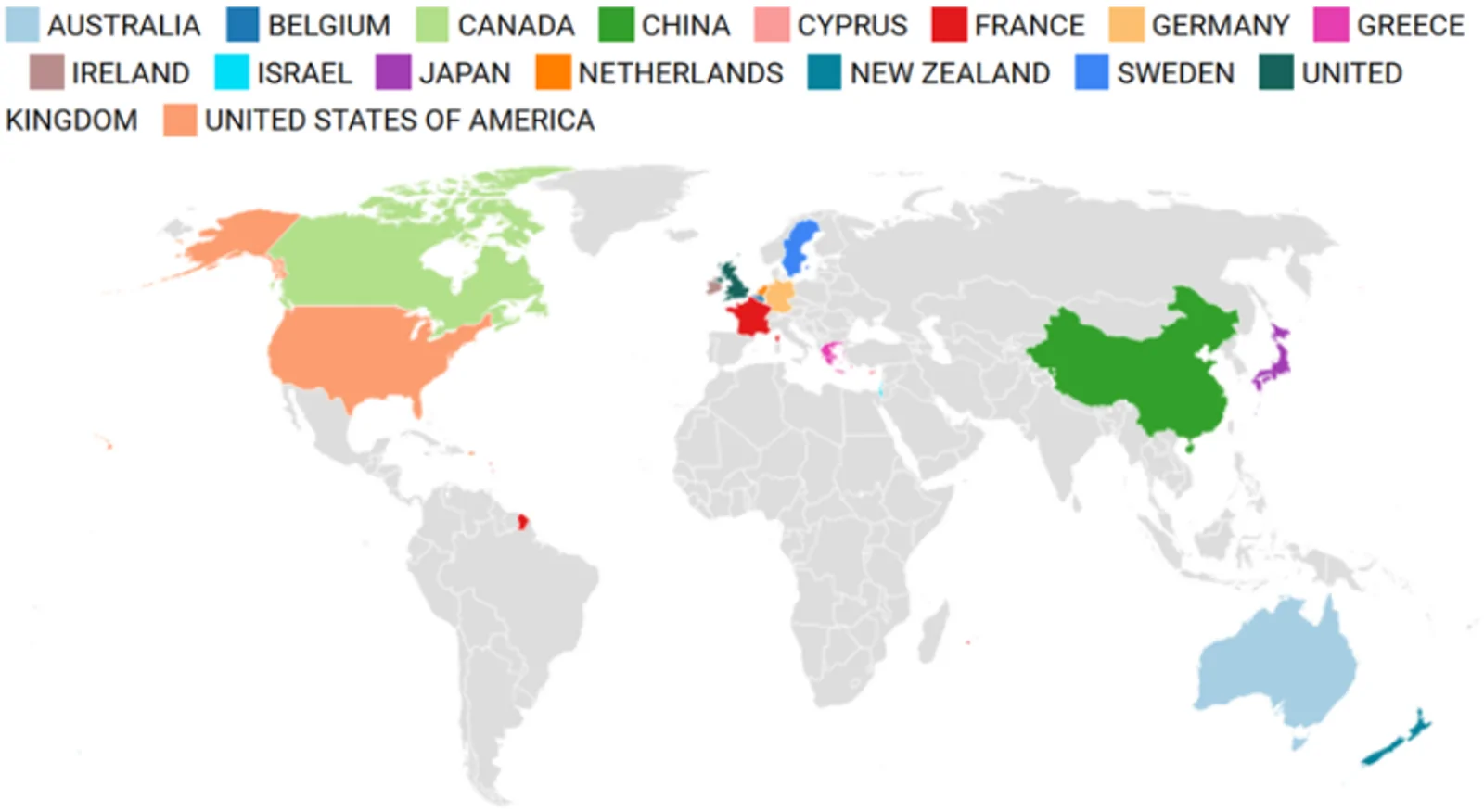
Geographical distribution of included studies

The included studies (*n* = 21) were conducted in Europe (*n* = 8), North America (*n* = 7), Asia (*n* = 3), and Oceania (*n* = 3). One European study was conducted across multiple countries (Cyprus, France, Greece, Ireland, and the United Kingdom). The geographical distribution of studies is illustrated in Fig. 2.

**Fig. 3:**
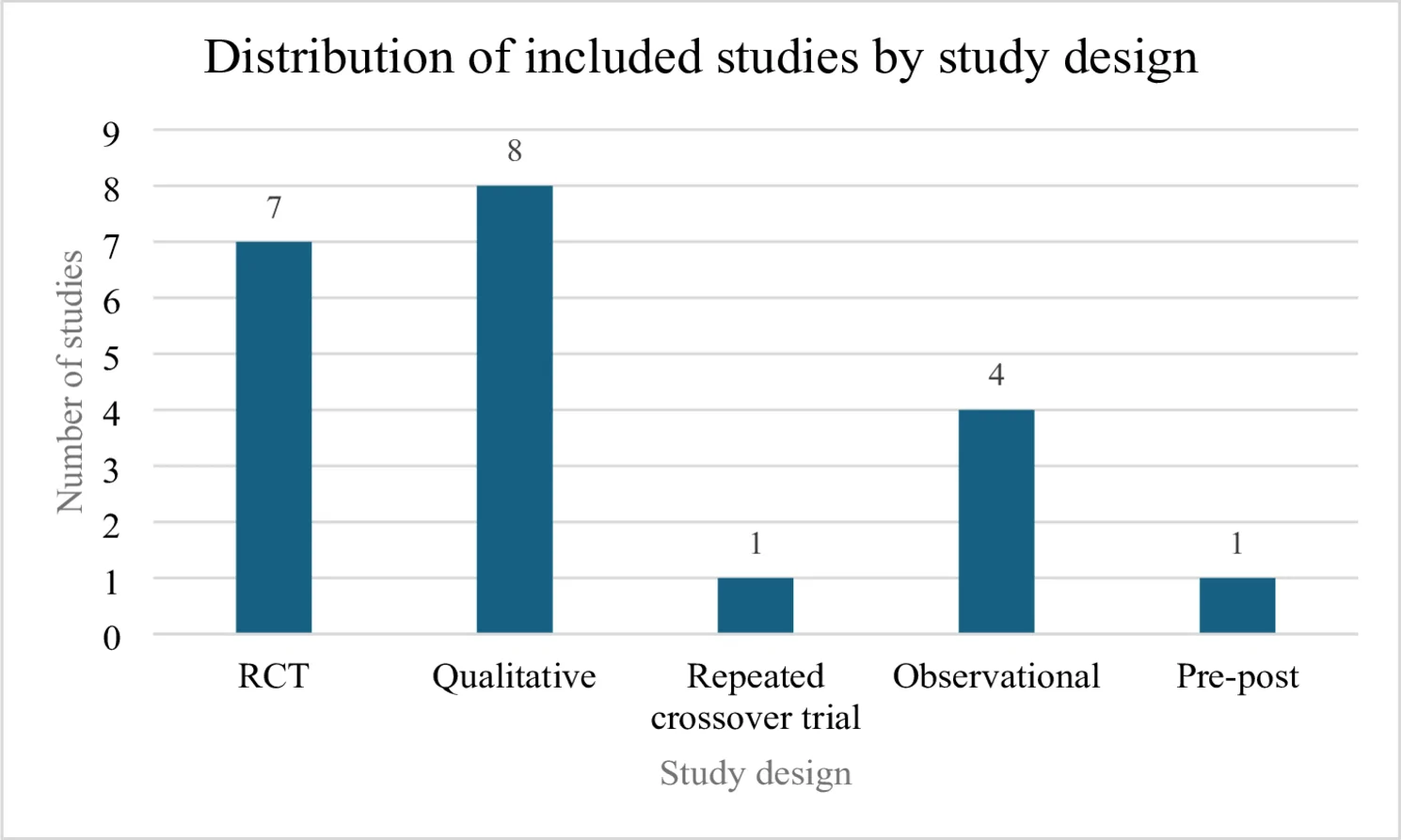
Distribution of included studies by study design. Note: Studies were classified by primary study design

The included studies comprised seven randomised controlled trials [38–44], one participant-blinded repeated crossover trial incorporating in-depth interviews [20], eight qualitative studies [22, 24, 45–50], one prospective observational mixed-methods study [23], one prospective single-arm observational study [51], one longitudinal observational analysis nested within a cluster-randomised trial cohort [52], one prospective pre–post study [53], and one longitudinal cohort study [54]. The distribution of study designs is presented in Fig. 3.

### Participant characteristics

The included samples were predominantly older, with most studies involving participants aged 65 years or older. The care and support contexts represented across the studies included hearing rehabilitation and communication support, dementia care, dual-sensory rehabilitation, community-delivered hearing care, and family- or care-partner-supported management of hearing difficulties. Several qualitative studies also included family members, care partners, or hearing-care professionals to capture relational and care-context perspectives.

Four studies included people with cognitive impairment or dementia and hearing difficulties [22, 38, 39, 52] and two involved participants with dual sensory impairment [41, 45]. The remaining studies primarily involved adults with hearing loss or hearing difficulties in hearing-care, rehabilitation, or contexts involving HAT use [20, 23, 24, 40, 42–44, 46–51, 53, 54]. Detailed population characteristics and the care and support context documented in each study are presented in Additional File 1.

#### Hearing assistance provision and technologies

The included studies examined a range of HATs and modes of provision. Conventional HAs formed the technological basis of several intervention and observational studies, including studies of HA provision, first-time fitting, and existing HA use [20, 38, 42, 51–54]. Hearing-care interventions also combined amplification or existing HA use with counselling, communication support, rehabilitation, or self-management support [40, 43, 44]. Smartphone-connected or app-based auditory technologies were investigated in two studies [23, 46], while multicomponent sensory-rehabilitation programmes incorporating hearing support were evaluated in people with dementia or dual sensory loss [39, 41]. Several qualitative studies additionally examined experiences of existing HA use and hearing-care contexts [22, 24, 47–50]. Detailed intervention and exposure characteristics are presented in Additional File 1.

### Social health outcomes

Findings relevant to one or more domains of social health were identified across the included studies and mapped deductively onto the multidimensional social health framework proposed by Vernooij-Dassen and colleagues [17]. This approach enabled structured comparison across heterogeneous study designs and outcome measures. Substantial heterogeneity was observed in terminology, measurement instruments, and the operationalisation of social-health constructs. Findings were derived from quantitative measures, hearing-specific questionnaires, and qualitative data. Given the diversity in study design and outcome measurement, findings were synthesised descriptively to map the breadth, distribution, and characteristics of the available evidence.

**Fig. 4:**
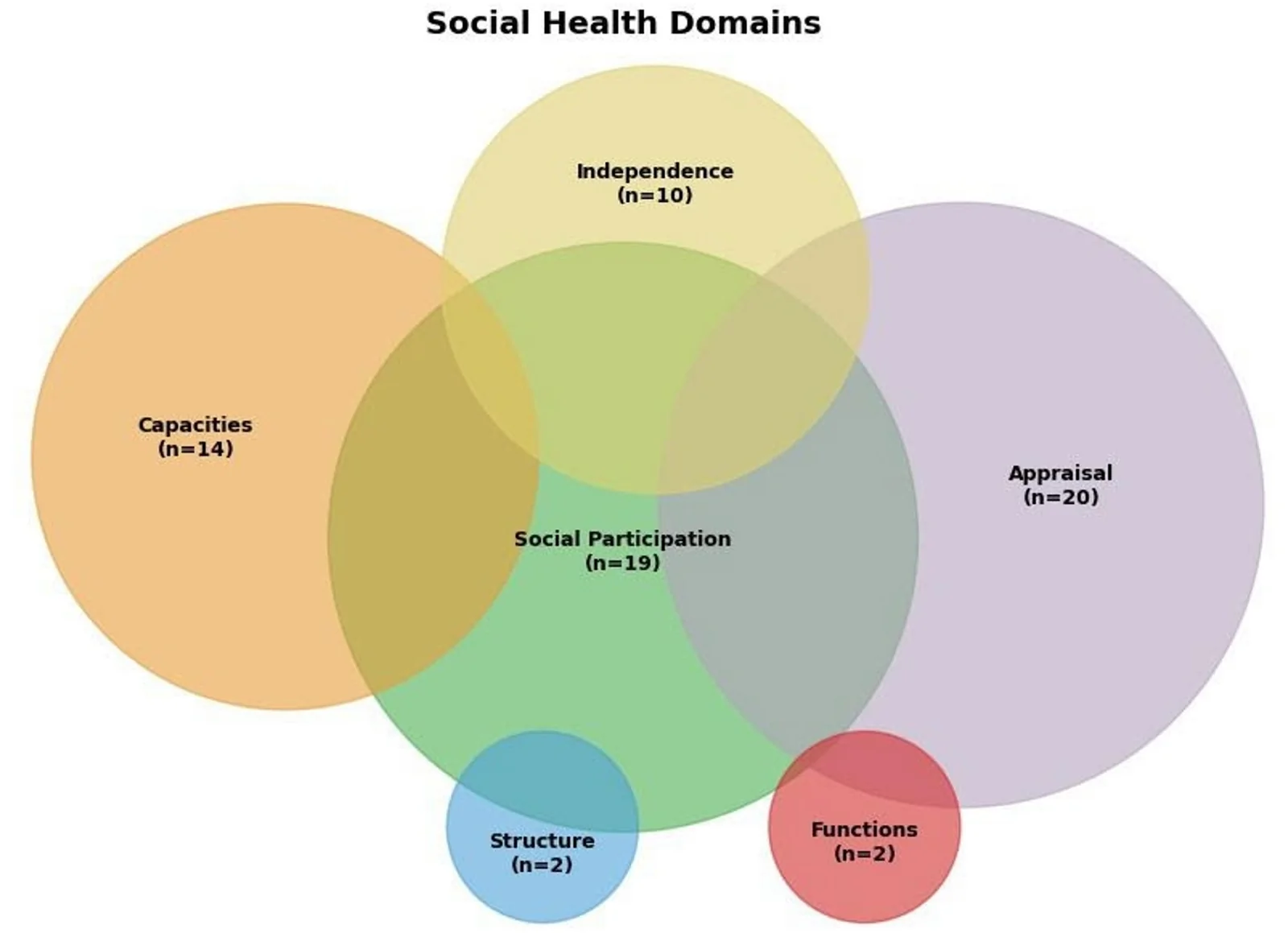
Distribution of included studies across social health domains

The findings were organised into individual-level and social-environmental domains. The individual level comprised Capacity, Independence, and Social Participation, while the social-environmental level comprised Structure, Function, and Appraisal. Table 3 summarises the conceptual definitions, studies contributing findings to each domain, and corresponding measures and data sources. The distribution of included studies across these domains is illustrated in Fig. 4

**Table 3:** Mapping of findings across domains of the social health framework

	Domain	Conceptual Definition	Studies contributing findings relevant to the domain	Measures and data sources
Individual level domains	Capacity	Ability to fulfil social potential and obligations	[20, 22–24, 39–41, 43, 44, 46, 48–51]	CSS; Listening Self-Efficacy Questionnaire; Ida Institute Line; HHIE/HHIE-S; TMIG-IC; qualitative interview data
	Independence	Autonomy in managing everyday life	[22–24, 38, 39, 42, 45, 46, 51, 54]	IADL, ADL, BADL, TMIG-IC, qualitative interview data
	Social Participation	Involvement in social activities and interactions	[20, 22–24, 38–51, 54]	HHIE/HHIE-S social or situational content; IOI-HA participation-related items; Duke Health Profile; self-reported social-activity measures; qualitative interview data
Social-environmental domains	Structure	Size, composition, and pattern of social ties	[43, 51]	Social Network Index; Convoy Model for Social Relationships
	Function	Support and exchanges provided through relationships	[49, 51]	Convoy Model for Social Relationships; qualitative interview data
	Appraisal	Perceptions and evaluations of the quality of relationships and interactions	[20, 23, 24, 38–54]	Relationship Satisfaction Scale; UCLA Loneliness Scale; De Jong Gierveld Loneliness Scale; HHIE/HHIE-S; ADRQL; QoL-AD; DEMQoL/DEMQoL-Proxy; qualitative interview data

Note: Studies could contribute findings to more than one domain. Study counts indicate findings interpreted in relation to each domain and do not imply direct measurement of the domain. Multidimensional measures were interpreted with reference to relevant item/subscale content where available. Measures are listed as data sources contributing domain-relevant findings; inclusion does not imply that the full instrument directly measured the corresponding domain

ADL: Activities of Daily Living; ADRQL: Alzheimer’s Disease Related Quality of Life; **BADL**: Bristol Activities of Daily Living; CSS: Communication Strategies Scale; DEMQoL/DEMQoL-Proxy: Dementia Quality of Life Instrument; HHIE/HHIE-S: Hearing Handicap Inventory for the Elderly/Hearing Handicap Inventory for the Elderly – Screening Version; IADL: Instrumental Activities of Daily Living; IOI-HA: International Outcome Inventory for Hearing Aids; QoL-AD: Quality of Life in Alzheimer’s Disease; TMIG-IC: Tokyo Metropolitan Institute of Gerontology Index of Competence; UCLA Loneliness Scale: University of California, Los Angeles Loneliness Scale

Study counts within each domain indicate studies contributing findings interpreted in relation to that domain and should not be understood as the number of studies directly measuring the domain. Interpretation was guided by the original domain definitions. For multidimensional measures, relevant item- or subscale-level content was considered where available. Where only an overall score was reported, interpretation was limited to the construct represented by the measure and its conceptual relevance to the original domain definition.

### Individual level domains

#### Capacity

Fourteen studies contributed findings interpreted in relation to the Capacity domain [20, 22–24, 39–41, 43, 44, 46, 48–51]. Consistent with the original framework, Capacity was understood as the ability to fulfil social potential and obligations [17]. Communication competence and socially situated communication skills were interpreted in relation to Capacity where they reflected functioning within everyday social roles or interactions [19].

Capacity-related findings centred on communication strategies and communication competence. Findings concerning listening confidence, self-efficacy, hearing-related functioning, and perceived control over communication or listening situations were interpreted cautiously in relation to Capacity and were not considered equivalent to the domain itself. Among intervention studies, Malmberg et al. reported greater improvements in communication-strategy scores following online hearing support [40], while Nieman et al. reported improvements in self-perceived communication function and listening self-efficacy [43]. The Baltimore HEARS pilot also reported improvements in hearing-related communication outcomes [44].

Participant-reported and qualitative findings further described increased confidence in everyday communication, strategies for managing challenging listening situations, and greater perceived control over hearing technologies [20, 22–24, 46]. Smartphone-connected and self-adjustable technologies were also described in relation to greater control over device settings and communication across different listening environments [23, 46]. These findings were interpreted as relevant to the conditions that may support social functioning.

#### Independence

Ten studies reported findings relevant to Independence, including autonomy, self-management, and the ability to manage everyday activities [22–24, 38, 39, 42, 45, 46, 51, 54]. Quantitative assessment included measures of activities of daily living, such as ADL, IADL, BADL, and the TMIG-IC, alongside qualitative accounts of self-management and everyday functioning.

No significant between-group or longitudinal changes were reported in ADL, IADL, BADL, or related measures of daily functioning [38, 39, 42, 54].

Qualitative studies described self-management and continued engagement in everyday activities and roles [24, 45], while studies of smartphone-connected or self-adjustable technologies also described greater perceived control over listening environments and device management [23, 46]. Findings concerning technology management were interpreted in relation to Independence only where they reflected autonomy or the management of everyday life. Hooper et al. additionally described circumstances in which memory difficulties and challenges with device handling increased reliance on care-partner support [22].

#### Social participation

Nineteen studies reported findings relevant to Social Participation, including engagement in social activities and participation in family, community, or work-related contexts, as well as communication-related restrictions affecting participation [20, 22–24, 38–51, 54]. Participation-related findings were assessed using hearing-specific questionnaires, measures of social activity, and qualitative accounts of involvement in everyday social situations.

Quantitative participation-related findings varied across studies. Ye et al. reported reductions in hearing-related situational restrictions following HA provision [42]. In the Baltimore HEARS pilot, 80% of participants reported feeling more connected to others following the intervention [44]. Malmberg et al. reported improvements in several hearing-related and communication-strategy outcomes, although the HHIE Social subscale did not show a significant between-group difference [40]. Other studies reported no significant changes in social interaction or broader social engagement [38, 54].

Qualitative and mixed-methods studies described HAT use as supporting day-to-day conversational involvement and continued participation in routine social, family, and work-related activities [20, 22–24, 45–47]. Contextual barriers were also described, including stigma, avoidance of social situations, and reduced participation associated with concerns regarding device visibility or social identity [48–50].

### Social environmental level domains

#### Structure

Two studies reported findings relevant to structural characteristics of social networks, including network size, composition, and kinship ties [43, 51]. Nieman et al. found no significant between-group change in broader social-network measures following the intervention [43]. Uchida et al. observed no change in overall social-network size over six months, while the number of kin contacts increased [51].

#### Function

Evidence relating to the Function of social relationships was limited. Two studies contributed findings concerning support within close relationships [49, 51]. Nickbakht et al. described supportive communication adjustments by family members, alongside dismissive or critical responses to hearing difficulties [49]. Uchida et al. reported improvement in family/relative-related HHIE items following HA fitting [51].

#### Appraisal

Findings interpreted in relation to the Appraisal domain were identified in twenty studies [20, 23, 24, 38–54]. Consistent with the original framework, Appraisal concerned individuals’ perceptions and evaluations of the quality of relationships and social interactions [17, 19].

Quantitative findings varied across studies. Leroi et al. found no significant between-group improvement in relationship satisfaction at the primary follow-up [39]. Nieman et al. reported no significant between-group differences in loneliness scores [43]. In the Baltimore HEARS pilot, loneliness scores did not change significantly, although 80% of participants reported feeling more connected to others and 67% reported feeling less lonely following the intervention [44]. In the prospective pre–post study by Weinstein et al., perceived loneliness decreased significantly following first-time HA fitting [53].

Broader quality-of-life findings were also reported and were interpreted in relation to the construct and content of the respective measures rather than as specific indicators of Appraisal. In the randomised trial by Adrait et al., ADRQL scores were higher at 12 months in the group initially fitted with active HAs [38]. In the longitudinal observational analysis by Atef et al., HA use was associated with higher QoL-AD scores at one year, although this association was not sustained at two years [52].

Stigma-related findings were interpreted in relation to Appraisal where they concerned perceived judgement or evaluation within social interactions. Qualitative studies described stigma and social evaluation in relation to hearing loss and HAs, including embarrassment or shame, concealment or non-disclosure, perceived judgement, and distancing or reduced engagement in some social situations [45, 47–50]. Smartphone-connected or app-based technologies were sometimes perceived as more socially acceptable or less stigmatising in everyday settings [23, 46]. In Maidment et al., some participants described conventional HAs as “not very cool,” whereas smartphone-based devices were perceived as more acceptable in everyday use because smartphone use was already embedded in daily life [46].

## Discussion

This scoping review mapped how HATs have been examined in relation to social health among individuals with care and support needs. Most included studies involved predominantly older samples, with evidence concentrated in the Appraisal and Social Participation domains and comparatively limited attention to the Structure and Function of social relationships. Applying the multidimensional social-health framework provided a structured basis for organising heterogeneous findings across distinct but interrelated dimensions of social health and highlighted domains that remain comparatively underexamined.

Appraisal-related findings most commonly concerned loneliness and relationship satisfaction, while stigma and perceived social judgement were relevant where they reflected how social relationships or interactions were perceived and experienced. Qualitative studies described embarrassment, concealment, anticipated judgement, and concerns about the social meaning of hearing loss and HAs [47–50]. These findings are consistent with previous research indicating that hearing-related stigma may shape disclosure, help-seeking, technology use, and participation in everyday interactions [8, 55]. Studies of smartphone-connected and app-based technologies also suggested that familiar consumer-device formats may be perceived as less conspicuous or less stigmatising in some social settings [23, 46]. Perceptions of hearing technologies therefore appear to be shaped by interpersonal context and prevailing social norms as well as by device characteristics.

Loneliness and social isolation are related but conceptually distinct aspects of social health. Loneliness primarily reflects subjective perceptions of social relationships, whereas social isolation relates more closely to the extent and structure of social connections [13, 27, 28]. This distinction is also reflected in the multidimensional social-health framework, in which loneliness aligns more closely with Appraisal and social isolation with Structure [17, 19].

In the included studies, subjective experiences such as loneliness and perceived connectedness were examined more frequently than structural characteristics of social networks. These findings further illustrated that subjective social experience and structural aspects of social connection may not change in parallel [43, 44]. Quality-of-life findings were mixed across studies. Adrait et al. reported higher ADRQL scores at 12 months in the group initially fitted with active HAs [38], whereas Leroi et al. found no significant between-group improvement in DEMQoL or relationship satisfaction at the primary follow-up [39]. In the longitudinal observational analysis by Atef et al., higher QoL-AD scores among HA users at one year were not sustained at two years [52]. Overall, changes in quality-of-life measures were not consistently accompanied by changes in more specific relational outcomes.

Social Participation was also widely represented in the included evidence. Hearing-specific measures and qualitative accounts described changes in communication-related restrictions and involvement in everyday social situations [20, 22–24, 40, 42, 44–47]. Ye et al. reported reductions in hearing-related situational restrictions following HA provision [42], while participants in the Baltimore HEARS pilot reported improvements in perceived communication and connectedness [44]. By contrast, no significant changes were reported in social interaction or social engagement in Adrait et al. and Dawes et al. [38, 54]. Participation is shaped by factors extending beyond auditory access, including environmental accessibility, established social routines, health and functional circumstances, opportunities for interaction, and interpersonal support [14]. HATs may therefore reduce communication-related barriers without necessarily producing corresponding changes in the frequency or pattern of social participation.

Capacity-related findings centred primarily on communication strategies and communication competence, with related findings concerning listening confidence, self-efficacy, and perceived control in everyday communication. Improvements in communication strategies were reported following online hearing support [40], while a community-delivered hearing-care intervention improved self-perceived communication function and listening self-efficacy [43]. Qualitative studies similarly described confidence, communication strategies, and perceived control in managing hearing technologies and challenging listening situations [22–24, 46]. These findings are consistent with previous evidence linking amplification with listening self-efficacy [56]. Communication-related competencies may contribute to the ability to fulfil social potential and obligations, although their relevance to Capacity depends on how they are situated within everyday social roles and interactions.

The use of hearing technologies was also shaped by the circumstances in which technologies were introduced and used. Studies informed by behavioural frameworks identified influences related to capability, opportunity, and motivation, alongside device usability, digital skills, access to support, and the demands of everyday communication environments [23, 46]. Hearing-care experiences are also influenced by fitting and follow-up processes, service accessibility, and the perceived suitability of the technology for an individual’s communication priorities [26, 57]. The incorporation of HATs into everyday life therefore depends on the fit between the individual, the technology, the service context, and the environments in which communication takes place rather than on individual capability or motivation alone.

Independence was examined less frequently. Quantitative studies assessing activities of daily living or related functional measures generally did not identify significant changes following hearing interventions or in association with HA use [38, 39, 42, 54]. Qualitative evidence, however, described self-management, perceived control over listening environments, and continued involvement in everyday activities [22–24, 45, 46]. At the same time, difficulties with device handling, maintenance, memory, and adjustment could increase reliance on family members or care partners [22]. Such reliance does not necessarily indicate diminished autonomy; support from others may facilitate continued involvement in communication, everyday activities, and decision-making. This is consistent with an interdependent understanding of autonomy in which agency can be maintained through appropriate relational support [6, 7].

The co-occurrence of hearing and cognitive difficulties is particularly relevant in later life and in care contexts. Several included studies involved people with cognitive impairment or dementia receiving hearing support, sensory rehabilitation, or dementia care [22, 38, 39, 52]. Cognitive changes may affect conversational processing, remembering device routines, and handling equipment, while hearing difficulties may further complicate communication with family members, care partners, and professionals [22, 58, 59]. Care-partner involvement may therefore become increasingly important for communication and device management, while also shaping patterns of support and decision-making. Social-health-related findings in these populations are best interpreted within the combined hearing, cognitive, interpersonal, and care context rather than in relation to hearing technology alone.

Patterns of device use were also relevant to the interpretation of social-health-related findings. Frequency or continuity of use does not necessarily indicate whether use is meaningful or aligned with an individual’s preferences [57, 60]. Decisions about when and where to use hearing technologies may be influenced by sound quality, comfort, usability, visibility, affordability, communication demands, access to follow-up care, and the extent to which the technology corresponds with the user’s priorities [23, 24, 57, 60, 61]. Intermittent use or non-use may therefore reflect limitations in technological, service, or contextual fit rather than insufficient motivation. Future studies would benefit from examining not only duration or frequency of use but also the situations in which technologies are used, avoided, or adapted and how these patterns relate to users’ own communication and participation goals.

Structure and Function were the least represented domains in the included evidence. Only a small number of studies examined social-network characteristics or supportive exchanges within relationships [43, 49, 51]. This gap is important because loneliness does not capture the size, composition, frequency of contact, or supportive functions of social networks. Social relationships may also be organised differently across cultural, family, and community contexts. For example, the prominence of whānau relationships, cultural participation, and intergenerational roles in the study by Manuel et al. illustrates forms of social connection that may not be fully represented by conventional individual-level measures [47]. Greater use of network-sensitive and culturally responsive approaches may therefore provide a more comprehensive understanding of how hearing technologies intersect with social relationships and support systems.

From a geriatric care perspective, the predominance of older participants in the included evidence makes these findings particularly relevant to services supporting people with multiple communication, functional, or cognitive needs. HATs are unlikely to operate as stand-alone technical solutions in such contexts. Accessible technology design, appropriate fitting and follow-up, communication support, care-partner involvement, and responsive service organisation may all influence how technologies are incorporated into everyday life [11, 22, 39, 44]. Person-centred hearing care should therefore consider auditory needs alongside communication priorities, preferred patterns of technology use, available support, and the social environments in which communication occurs.

### Limitations

Several limitations should be considered when interpreting these findings. Although studies from Europe, North America, Asia, and Oceania were included, the evidence was concentrated in high-income countries, and no eligible studies were identified from Africa or Latin America. Publications were restricted to English, German, and Turkish, and relevant evidence published in other languages may therefore not have been captured. The geographical and linguistic distribution limits the global representativeness of the mapped evidence.

Substantial heterogeneity was observed in study design, participant characteristics, technologies, outcome measures, and follow-up duration. Social-health-related findings were derived from hearing-specific questionnaires, multidimensional measures, social-network measures, and qualitative data, limiting direct comparison across studies. In addition, the included studies did not consistently conceptualise or measure social health according to the multidimensional framework applied in this review. Mapping therefore required interpretive judgement, particularly for multidimensional measures and findings relevant to more than one domain. The framework was used as a conceptual structure for organising the evidence rather than as a model to be tested or validated.

The evidence base included randomised trials, observational studies, pre–post studies, and qualitative research. Associations and longitudinal changes observed in non-randomised studies cannot establish effects attributable to HAT use. Consistent with the purpose of a scoping review, formal critical appraisal and quantitative synthesis were not undertaken. The findings should therefore be interpreted as a mapping of the extent, characteristics, and distribution of the available evidence rather than as estimates of intervention effectiveness or certainty of effect.

## Conclusion

Among the studies included in this review, evidence concerning HATs and social health was concentrated in the Appraisal and Social Participation domains and was derived predominantly from older samples. Communication-related capacity and participation findings were commonly reported, whereas considerably fewer studies examined social-network Structure or the supportive Function of relationships. Findings concerning social interaction, social engagement, and relational outcomes varied across studies and were closely situated within interpersonal, environmental, technological, and service contexts.

Applying the multidimensional social-health framework provided a structured means of organising the heterogeneous evidence and identifying domains that remain comparatively underexamined. Future research should incorporate network-sensitive and culturally responsive measures, and account for co-occurring cognitive and communication difficulties. Greater attention to accessible technology design, service organisation, communication environments, patterns of meaningful or preferred use, and individuals’ own priorities may provide a more comprehensive understanding of how HATs relate to social health among individuals with care and support needs.

## Supplementary Information

Below is the link to the electronic supplementary material.

Supplementary Material 1

Supplementary Material 2

## Data Availability

All data generated or analysed during this study are included in this published article and its additional files. Additional data are available from the corresponding author on reasonable request.

## Abbreviations

ADL: Activities of Daily Living
ADRQL: Alzheimer’s Disease Related Quality of Life
BADL: Bristol Activities of Daily Living
HATs: Hearing Assistance Technologies
HHIE: Hearing Handicap Inventory for the Elderly
IADL: Instrumental Activities of Daily Living
IOI-HA: International Outcome Inventory for Hearing Aids
OSF: Open Science Framework
PCC: Population–Concept–Context
PRISMA-ScR: Preferred Reporting Items for Systematic Reviews and Meta-Analyses Extension for Scoping Reviews
QoL-AD: Quality of Life in Alzheimer’s Disease
TMIG-IC: Tokyo Metropolitan Institute of Gerontology Index of Competence
