# Supplementary material for: Hearing assistance technologies and social health among individuals with care and support needs: a scoping review
Source: BMC Geriatr. 2026 Nov 10;26:1401. doi: 10.1186/s12877-026-08681-3 (PMC13749917; doi:10.1186/s12877-026-08681-3)
Supplement: Supplementary file 1 [file 12877_2026_8681_MOESM1_ESM.docx]

**Additional File 1. Characteristics of included studies (n = 21)**

| Study | Country | Study design | Aim | HAT / intervention or exposure | Population and basis for care/support relevance | Key findings | Measures / data sources | Findings relevant to social health |
| --- | --- | --- | --- | --- | --- | --- | --- | --- |
| Adrait et al. (2017) | France | Multicentre double-blind RCT with semi-crossover (12 months) | Assess the effects of binaural HAs on neuropsychiatric symptoms, IADL, QoL, social interaction, and compliance | Binaural active HAs vs placebo; placebo devices activated at 6 months | 51 adults with ARHL and Alzheimer’s disease were randomised; 48 were fitted with HAs (active HA: n = 22, mean age 83.0; placebo: n = 26, mean age 82.3); dementia and hearing-care context with caregiver-reported outcomes | No significant changes at 6 months; ADRQL scores were higher in the active-HA group, and better outcomes were associated with greater HA compliance | NPI; IADL; ADRQL; Zarit Burden; Duke Health Profile; HA compliance | No significant change in social interaction or IADL; ADRQL scores improved at 12 months |
| Atef et al. (2023) | Germany | Longitudinal observational analysis nested within a cluster-randomised trial cohort (2-year follow-up) | Examine associations between HA use and longitudinal changes in cognition, depressive symptoms, and QoL among people with cognitive impairment or dementia | HA use as an observational exposure; HA users vs non-users | 258 community-dwelling adults aged ≥70 years with cognitive impairment or dementia (mean age 79.9) from the GP-based DelpHi-MV dementia-care study; 123 reported HL and 54 used HAs | Among participants with HL, HA use was associated with a smaller increase in depressive symptoms over 2 years and higher QoL-AD scores at 1 year, but not at 2 years; no association with cognitive decline was observed | MMSE; GDS; QoL-AD | Higher QoL-AD scores were observed at 1 year; only total QoL-AD scores were reported |
| Cox et al. (2016) | USA | Participant-blinded repeated crossover trial incorporating in-depth interviews | Compare patient-reported outcomes of premium- and basic-feature HAs during everyday use | Four bilaterally fitted HA models (premium and basic technology from two manufacturers) | 45 adults aged 61–81 years (mean age 70.3) with mild-to-moderate bilateral hearing loss; each identified at least one specific everyday listening situation in which hearing help was desired | All four HA models provided substantial perceived benefit in daily life; no significant differences were found between premium- and basic-feature devices in patient-reported outcomes or overall preferences | Hearing-related QoL change; six-item everyday-hearing questionnaire; in-depth interviews; device-preference ratings | Participants reported benefits in everyday hearing and communication across devices; premium technology conferred no additional perceived benefit |
| David et al. (2018) | Israel | Qualitative study | Examine self-stigma among older adults with ARHL | HAs and hearing devices discussed in relation to stigma; no study intervention | 11 older adults with ARHL (mean age 80.7); interviews examined hearing-related self-stigma expressed through concealment, social distancing, and adaptation to hearing aids, providing a relational and compensatory-support context relevant to everyday communication and social interaction | Participants described self-stigma through stereotypes of ageing and disability, shame or ridicule, concealment, distancing, and adaptation to HAs | In-depth semi-structured interviews | Stigma was reflected in concealment and distancing from social situations |
| Dawes et al. (2015) | USA | Longitudinal cohort study | Examine long-term outcomes associated with HA use | Existing conventional HA use as an observational exposure | 666 community-dwelling adults aged 48–92 years with hearing impairment at a level expected to result in communication disability and indicate candidacy for hearing support; hearing handicap (HHIE-S), daily functioning, and social engagement were assessed longitudinally, with HA use examined as an observational exposure | HA users and non-users did not differ in social engagement, cognition, or mental health across follow-up; aided HHIE-S scores were better, and HA users had better SF-12 physical health at 11 years | HHIE-S; SF-12; MMSE; Trail Making; AVLT; ADL/IADL; social engagement metrics | HA use was not associated with social engagement or IADL |
| Gomez et al. (2022) | UK | Single-centre prospective observational mixed-methods study; qualitative Phase II reported | Identify patient-reported barriers and facilitators to smartphone-connected HA use and app-based self-management | 7-week trial of bilateral smartphone-connected HAs controlled through a prototype app | 8 HA users aged 65–81 years (mean age 71.75), recruited from NHS Adult Audiology Services after hearing assessment; 1 first-time and 7 existing HA users received HA fitting, counselling, app instruction, and follow-up | Participants described smartphone-connected HAs as supporting self-management, confidence, and participation; digital literacy, decisional burden, social norms, and perceived stigma influenced use | Focus groups; thematic analysis informed by COM-B, TDF, and the Behaviour Change Wheel | Everyday communication, participation, confidence, and stigma were prominent in participants' experiences |
| Hooper et al. (2024) | UK | Qualitative study | Explore HA use among people with dementia and their care partners | HA use following sensory-support intervention; no additional intervention in the qualitative study | 11 person-with-dementia/care-partner dyads (people with dementia: median age 80, range 72–89); care partners were involved in HA routines and support | Care-partner support, routines, and practical adaptations facilitated HA use, whereas memory, device-management, and communication difficulties hindered sustained use | TDF-based semi-structured interviews | Care-partner involvement supported communication and participation; reliance on support was also described |
| Jaiswal et al. (2020) | Canada | Qualitative study | Explore barriers and facilitators of social participation in dual sensory impairment | Assistive devices, including HAs, discussed as facilitators; no study intervention | 16 adults aged ≥65 years with DSI (mean age 86), registered with an urban sensory rehabilitation organisation | Assistive devices and social support facilitated day-to-day functioning and social participation; inaccessible environments, transportation barriers, cost, stigma, and negative attitudes restricted participation | ICF-based semi-structured interviews, content analysis | Assistive devices and social support facilitated communication, participation, and independence; stigma related to visible device use was also described |
| Johnson et al. (2024) | USA | Qualitative study | Explore experiences of successful HA users | Existing successful HA use; no study intervention | 5 older adults aged 66–83 years with high hearing-aid self-efficacy; interviews explored strategies and sources of support contributing to successful rehabilitation and sustained HA use | Participants attributed successful HA use to intrinsic motivation, self-reliance in health decisions, advice from trusted others, pro-social attributes, positive expectations, and active pursuit of optimal HA outcomes | Semi-structured interviews informed by the Transtheoretical Model of Behaviour Change | Trusted others, motivation, and self-reliance supported sustained HA use |
| Leroi et al. (2024) | Cyprus, France, Greece, Ireland, UK | Multicentre observer-blind parallel-group RCT | Evaluate a home-based sensory intervention for people with dementia | 18-week home-based Sensory Support Intervention (HAs and vision support) vs usual care | 252 adults with mild-to-moderate dementia and adult-acquired hearing and/or vision difficulties (mean age 79.6); home-based sensory support vs care as usual, with care-partner involvement | The intervention did not improve the primary DEMQoL outcome at 36 weeks; no significant between-group differences were found in functional ability, sensory-related functioning, neuropsychiatric symptoms, or relationship satisfaction at the primary follow-up | DEMQoL; BADL; VALV-VFQ; HHIE; MoCA; NPI; Relationship Satisfaction Scale | No significant between-group differences were observed in relationship satisfaction or DEMQoL at the primary follow-up |
| Maidment et al. (2019) | UK | Qualitative study following a 2-week real-world device trial | Explore experiences of smartphone-connected listening devices | 2-week trial of one smartphone-connected listening device: made-for-smartphone HA, PSAP, smartphone hearing-aid app with wired earphones, or wireless hearable | 20 adults with mild-to-moderate hearing loss (mean age 62.25; range 21–81), all experienced conventional HA users; unaided HHIE scores documented hearing-related handicap, and participants trialled smartphone-connected listening devices in everyday life to address individual listening and communication needs | Participants described fine-tuning and personalisation as supporting autonomy, empowerment, and control over hearing loss; sound quality, comfort, digital skills, portability, cost, and stigma shaped device use | Individual semi-structured interviews; COM-B-informed deductive thematic analysis | Participants linked self-adjustment with confidence and everyday participation; smartphone-based formats were sometimes perceived as less stigmatising |
| Malmberg et al. (2023) | Sweden | Parallel-group RCT | Evaluate online support for HA self-management | 5-week online hearing-support programme vs standard care | 136 HA users (75% aged 65–95 years); participants received structured online hearing support or standard care | Online hearing support produced greater improvement in HHIE total and Emotional subscale scores and in CSS total, Verbal, and Nonverbal subscale scores; the HHIE Social subscale did not differ significantly between groups | HHIE; CSS; self-report of hearing problems and communication competence | Communication strategies improved; the HHIE Social subscale showed no significant between-group difference |
| Manuel et al. (2025) | New Zealand | Qualitative study | Explore Māori experiences of HL and hearing healthcare | Hearing-technology use and hearing-healthcare experiences; no study intervention | 12 kaumātua aged 60–89 years, 7 whānau members, 8 hearing-care professionals, and 6 Māori health professionals; whānau-centred hearing-care context | Kaumātua and whānau described hearing as central to connection, cultural participation, and intergenerational roles; stigma, access barriers, and non-whānau-centred services constrained hearing care, while whānau support and culturally safe care facilitated engagement | Interviews and focus groups | Whānau relationships, cultural participation, and intergenerational roles were central to hearing-care experiences; stigma and access barriers could restrict these experiences |
| Nickbakht et al. (2025) | Australia | Qualitative descriptive study | Explore experiences of stigma related to HL and HAs among adults with HL, family members, and hearing-care professionals | Existing HA use and HA-related stigma experiences; no study intervention | 20 community-dwelling adults with acquired HL aged 53–88 years, 20 nominated family members, and 25 hearing-care professionals; interviews addressed everyday communication, help-seeking, rehabilitation, and stigma | Participants associated HL and HAs with stereotypes of ageing, disability, and difference; stigma experiences varied across adults with HL, family members, and hearing-care professionals | Semi-structured interviews | Stigma was shaped by social identity, relationships, and ageing/disability stereotypes |
| Nieman et al. (2017) | USA | Pilot RCT with 3-month delayed-treatment wait-list control | Assess feasibility of a community-delivered hearing intervention | Community-delivered HEARS intervention with low-cost amplification and communication support | 15 participant–partner dyads (participant mean age 70.1) with mild-to-moderate HL and no prior HA use; intervention included amplification and communication support | HHIE-S and communication outcomes improved; loneliness and QoL did not change significantly; 80% reported feeling more connected and 67% reported feeling less lonely | HHIE-S; UCLA Loneliness Scale; SF-36; PHQ; Ida Institute Line; ATCQ; Quantified Denver; IOI-AI | Reduced hearing-related participation restrictions and improved perceived communication; participants also reported greater connectedness and less loneliness, although loneliness scores did not change significantly |
| Nieman et al. (2022) | USA | Open-label parallel group RCT with wait-list control | Evaluate a community-delivered low-cost hearing intervention | Community health worker-delivered hearing care including fitting/orientation to a low-cost personal sound amplification device and hearing rehabilitation | 151 adults (mean age 76.7); community-delivered hearing care addressed daily communication difficulties | At 3 months, the intervention improved HHIE-S communication function, listening self-efficacy, and the SF-12 physical component; loneliness, depression, social isolation, valuation of life, and technology-related self-efficacy did not differ significantly between groups | HHIE-S; SF-12; PHQ-9; UCLA Loneliness Scale; Social Network Index; Listening Self-Efficacy Questionnaire | Communication and listening self-efficacy improved; no change in loneliness or isolation |
| Scarinci et al. (2025) | Australia | Qualitative descriptive study | Explore responses to stigma related to HL and HAs | HL- and HA-related stigma responses; no study intervention | 20 adults aged >50 years with HL (mean age 69.4), 20 close family members, and 25 hearing-care professionals; interviews examined responses to stigma in everyday and hearing-care contexts | Participants described disclosure and non-disclosure of HL and HAs as context-dependent responses to stigma, alongside varied strategies for managing stigma in everyday life | Semi-structured interviews | Stigma shaped disclosure, interpersonal interaction, and help-seeking across social contexts |
| Uchida et al. (2021) | Japan | Multi-institutional prospective single-arm observational study | Examine changes in executive function and social interaction after HA adoption | Introduction of HAs with clinical fitting and follow-up; 6-month pre–post observation | 127 first-time HA users were enrolled; 94 completed 6-month follow-up (mean age 76.9), after recruitment from seven university HA clinics for hearing difficulty | At 6 months, DSST scores increased; overall social-network size was unchanged, while kin contacts increased and family/relative-related HHIE items improved | Audiometry; DSST; Convoy Model; HHIE; TMIG-IC | Network size was unchanged; kin contacts and family-related HHIE items improved |
| Vreeken et al. (2015) | Netherlands & Belgium | RCT | Evaluate an integrated dual sensory loss rehabilitation protocol | Dual Sensory Loss rehabilitation protocol delivered within low-vision rehabilitation | 128 adults aged ≥50 years with dual sensory loss (mean age 81.5); integrative rehabilitation protocol vs wait-list control | Non-significant overall improvement in IOI-HA outcomes; subgroup analyses indicated greater improvement among participants with baseline HA-use difficulties | IOI-HA; HAFQ; hearing and vision assessments | Subgroup analyses indicated reduced residual hearing-related difficulties among participants with greater baseline HA-use difficulties |
| Weinstein et al. (2016) | USA | Prospective pre–post study | Assess loneliness after first-time HA use | First-time bilateral digital HA fitting | 40 older adults aged 62–92 years with mild-to-moderately severe hearing loss, recruited from a private hearing practice after expressing a desire for hearing aids; participants underwent HA fitting and routine follow-up, with social and emotional loneliness assessed before and after fitting | Perceived loneliness decreased significantly after 4–6 weeks of HA use, with larger reductions among participants with moderate-to-severe HL | Gierveld Loneliness Scale; QuickSIN; Reading Span | Loneliness decreased following first-time HA fitting |
| Ye et al. (2022) | China | RCT | Assess the effects of HA provision on health outcomes | Free unilateral BTE HA provision with fitting and follow-up vs no-intervention control | 385 adults aged ≥45 years with clinically diagnosed moderate-or-greater hearing loss; baseline HHIE-S scores documented hearing-related handicap. The intervention provided free HA fitting together with adjustment, maintenance, and reinstruction, and social activity and social-network characteristics were assessed | Improved HHIE-S, reduced depressive symptoms; benefits limited to socially active participants; no changes in SF-12, ADL/IADL, MMSE | HHIE-S; SF-12; CES-D-10; MMSE; ADL; IADL; LSNS | Hearing-related situational restrictions improved; benefits were concentrated among participants with social activities or active social networks |

**Abbreviations:** ADL, Activities of Daily Living; ADRQL, Alzheimer’s Disease Related Quality of Life; ARHL, age-related hearing loss; ATCQ, Attitudes Toward Loss of Hearing Questionnaire; AVLT, Auditory Verbal Learning Test; BADL, Bristol Activities of Daily Living; BTE, behind-the-ear; CES-D-10, 10-item Center for Epidemiologic Studies Depression Scale; COM-B, Capability, Opportunity, Motivation–Behaviour; CSS, Communication Strategies Scale; DEMQoL, Dementia Quality of Life Instrument; DSI, dual sensory impairment; DSL, dual sensory loss; DSST, Digit Symbol Substitution Test; GDS, Geriatric Depression Scale; HA/HAs, hearing aid/hearing aids; HAFQ, Hearing Aid Fitting Questionnaire; HCP, hearing-care professional; HHIE/HHIE-S, Hearing Handicap Inventory for the Elderly/Screening Version; HL, hearing loss; IADL, Instrumental Activities of Daily Living; ICF, International Classification of Functioning, Disability and Health; IOI-AI, International Outcome Inventory–Alternative Interventions; IOI-HA, International Outcome Inventory for Hearing Aids; LSNS, Lubben Social Network Scale; MMSE, Mini-Mental State Examination; MoCA, Montreal Cognitive Assessment; NPI, Neuropsychiatric Inventory; PHQ/PHQ-9, Patient Health Questionnaire/9-item version; PSAP, personal sound amplification product; QoL, quality of life; QoL-AD, Quality of Life in Alzheimer’s Disease; QuickSIN, Quick Speech-in-Noise test; RCT, randomised controlled trial; SF-12/SF-36, 12-/36-item Short Form Health Survey; TDF, Theoretical Domains Framework; TMIG-IC, Tokyo Metropolitan Institute of Gerontology Index of Competence; UCLA, University of California, Los Angeles; VALV-VFQ, Veterans Affairs Low Vision–Visual Functioning Questionnaire.
