# Supplementary material for: Hearing assistance technologies and social health among individuals with care and support needs: a scoping review
Source: BMC Geriatr. 2026 Nov 10;26:1401. doi: 10.1186/s12877-026-08681-3 (PMC13749917; doi:10.1186/s12877-026-08681-3)
Supplement: Supplementary file 2 [file 12877_2026_8681_MOESM2_ESM.docx]

**Complete Electronic Search Strategy for PubMed**

**Database:** PubMed
**Date searched:** 9 April 2025
**Records retrieved:** 210
**Limits:** English, German, Turkish

**Platform:** PubMed (National Library of Medicine, NCBI)

**Publication-date limit:** No publication-date filter was applied during the electronic search; the predefined publication period was applied during eligibility assessment.

**Search strategy**

(("need for care"[Title/Abstract] OR "nurs*"[Title/Abstract] OR "care"[Title/Abstract] OR "caring"[Title/Abstract]) AND (("hearing aid*"[Title/Abstract] OR "hearing device*"[Title/Abstract] OR "hearing technolog*"[Title/Abstract] OR "hearing assistive device*"[Title/Abstract] OR "hearing assistive technolog*"[Title/Abstract] OR "assistive listening device*"[Title/Abstract] OR "hearable*"[Title/Abstract] OR "earable*"[Title/Abstract] OR "ear device*"[Title/Abstract] OR "ear sensor*"[Title/Abstract] OR "earbud*"[Title/Abstract] OR "ear-worn*"[Title/Abstract]) OR ("Hearing Aids"[Mesh]))) AND ("Social Health"[Title/Abstract] OR "social participation"[Title/Abstract] OR "social health"[Title/Abstract] OR "social isolation"[Title/Abstract] OR "social exclusion"[Title/Abstract] OR "social well-being"[Title/Abstract] OR lonel*[Title/Abstract] OR "community engagement"[Title/Abstract] OR "social engagement"[Title/Abstract] OR "capacity"[Title/Abstract] OR "reciprocity"[Title/Abstract] OR "independence"[Title/Abstract] OR "independent living"[Title/Abstract] OR "autonomy"[Title/Abstract] OR "activities of daily living"[Title/Abstract] OR "decision-making"[Title/Abstract] OR "self-management"[Title/Abstract] OR leisure activit*[Title/Abstract] OR meaningful activit*[Title/Abstract] OR social network*[Title/Abstract] OR "social environment"[Title/Abstract] OR "relationship quality"[Title/Abstract] OR "social relationship"[Title/Abstract] OR community network*[Title/Abstract] OR "social involvement"[Title/Abstract] OR "community involvement"[Title/Abstract] OR "social influence"[Title/Abstract] OR stigma*[Title/Abstract] OR "social interaction*"[Title/Abstract] OR "social control"[Title/Abstract] OR "social support"[Title/Abstract] OR social role*[Title/Abstract] OR social contact*[Title/Abstract] OR "social functioning"[Title/Abstract] OR "interpersonal relations"[Title/Abstract]) AND (english[Filter] OR german[Filter] OR turkish[Filter])
